# Relative gene expression of fatty acid synthesis genes at 60 days postpartum in bovine mammary epithelial cells of Surti and Jafarabadi buffaloes

**DOI:** 10.14202/vetworld.2017.467-476

**Published:** 2017-05-02

**Authors:** Mamta Janmeda, Vishnu Kharadi, Gaurav Pandya, Balkrishna Brahmkshtri, Umed Ramani, Kuldeep Tyagi

**Affiliations:** 1 Department of Animal Genetics and Breeding, College of Veterinary Sciences, Navsari Agricultural University, Navsari, Gujarat, India; 2 Livestock Research Station, Navsari Agricultural University, Navsari, Gujarat, India; 3 Department of Animal Biotechnology, College of Veterinary Sciences, Navsari Agricultural University, Navsari, Gujarat, India

**Keywords:** buffalo, gene expression, Jafarabadi, milk, Surti

## Abstract

**Aim:**

Aim of the study was to study the relative gene expression of genes associated with fatty acid synthesis at 60 days postpartum (pp) in bovine mammary epithelial cells (MECs) of Surti and Jafarabadi buffaloes.

**Materials and Methods:**

A total of 10 healthy Surti and Jafarabadi buffaloes of each breed were selected at random from Livestock Research Station, Navsari and Cattle Breeding Farm, Junagadh, Gujarat, respectively, for this study. Milk sample was collected from each selected buffalo at day 60 pp from these two breeds to study relative gene expression of major milk fat genes using non-invasive approach of obtaining primary bovine MECs (pBMEC) from milk samples.

**Results:**

In this study overall, the relative expression of the six major milk lipogenic genes butyrophilin subfamily 1 member A1 (*BTN1A1*), stearoyl-CoA desaturase *(SCD)*, lipoprotein lipase (*LPL*), glycerol-3-phosphate acyltransferase mitochondrial *(GPAM)*, acetyl-coenzyme A carboxylase alpha *(ACACA)*, and lipin *(LPIN)* did not show changes in expression patterns at 60^th^ day of lactation in both Surti and Jafarabadi buffaloes.

**Conclusion:**

The pBMEC can be successfully recovered from 1500 ml of milk of Surti and Jafarabadi buffaloes using antibody-mediated magnetic bead separation and can be further used for recovering RNA for down step quantification of major milk lipogenic gene expression. The relative expression of the six major milk lipogenic genes *BTN1A1*, *SCD*, *LPL*, *GPAM*, *ACACA*, and *LPIN* did not show changes in expression patterns in both Surti and Jafarabadi buffaloes, suggesting expression levels of lipogenic genes are maintained almost uniform till peak lactation without any significant difference.

## Introduction

Buffaloes are imperative sources of edible milk for human consumption in several parts of the world including India. The current buffalo population in India as per latest 19^th^ livestock census is 108.7 million which accounts for 21.23% of the total livestock population [1]. Gujarat had around 9.55% contemporary buffalo population of the country and bestowed with high milk producing breeds. Milk production in India grew at an annual growth rate of 5.0% and reached a volume of 127.9 million tons milk in the year 2011-2012 [2]. Buffalo was the prime contributor with 58.34% share to the total milk pool in Gujarat state [3]. Buffalo milk has long been valued by its important chemical composition determining nutritive properties and suitability in the manufacture of traditional as well as industrial dairy products. The composition of buffalo milk is mainly determined by fat, protein, solid not fat (SNF), and lactose. Buffalo milk is characterized by higher solids contents for being a richer source of lipids, protein, lactose, and minerals. Fat is the major energy source in milk, and lipid synthesis by the mammary glands is particularly impressive. In early lactation, daily milk fat secretion in the dairy cow can represent over 35% of net energy intake [4]. Rudolph *et al*. [5] had described the lactating mammary gland as “a lipid synthesizing machine.” Recently, due to the characterization of the lipogenic genes involved in milk synthesis and secretion as well as the development of molecular biology tools, few studies have been undertaken to relate the effects of milk fatty acid (FA) profile to mammary gland lipid metabolism [6].

Gene expression analysis is becoming more prevalent in livestock species since it promotes our understanding of complex biological processes such as lactation physiology [7,8]. In general, lactation is characterized by dramatic up-regulation in expression of lipogenic genes. Temporal expression of the genes with well-defined roles in mammary lipid metabolism peaked at 60 days postpartum and to some extent followed the lactation curve. The majority of genes in the fat metabolism pathway had high expression in transition and peak lactation milk [9]. Butyrophilin subfamily 1 member A1 (*BTN1A1)* is the most abundant milk fat globule membrane protein and it is produced in the end of pregnancy and maintained to the termination of the lactation [10]. Accumulating studies also showed *BTN1A1* played a key role in the formation and secretion of milk fat globules [11]. Stearoyl-CoA desaturase (*SCD*) is the important rate-limiting enzyme in biosynthesis of monounsaturated FAs and involved in the desaturation of FA [12]. Lipoprotein lipase (*LPL*) is an enzyme catalyzing the hydrolysis of triglyceride components of circulating chylomicrons and low-density lipoproteins [13]. *LPL* activity in mammary tissue increases markedly at parturition [14], and this increase may be important for the onset of milk fat secretion. Glycerol-3-phosphate acyltransferase mitochondrial (*GPAM*) is the enzyme whose transcribed protein catalyses the first step in the esterification of FAs to glycerol [15]. Acetyl-coenzyme A carboxylase alpha (*ACACA*) catalyses the rate-limiting step in *de novo* FA synthesis which produces SFA and palmitate from acetate [16]. Lactation results in a readjustment of lipid metabolism to meet synthetic demands of mammary gland for milk fat synthesis and this is associated with an increase in both activity and amount of mammary *ACACA* [17]. Lipin (*LPIN*) proteins are involved in dephosphorylation of phosphatidic acid to form diacylglycerol for the synthesis of triglycerides and phospholipids. This indicates that *LPIN* may have important function in milk fat synthesis. Genetic variability remained the prime focus of breeders to explore reasons for differential production potential among different breeds.

Surti and Jafarabadi are the two divergent breeds of buffalo in relation to body size, fat % and feed conversion efficiency. Keeping this in view, Surti and Jafarabadi buffalo breeds maintained at organized farms of state agricultural universities in their breeding tract were selected for this study. The experiment was designed to understand the relative gene expression of genes associated with FA synthesis at 60 days postpartum (pp) in bovine mammary epithelial cells (MECs) of Surti and Jafarabadi buffaloes.

## Materials and Methods

### Ethical approval

The prior approval from the Institutional Animal Ethics Committee was obtained for the use of Surti and Jafarabadi buffalo breeds maintained at Livestock Research Stations, Navsari and Cattle Breeding Farm, Junagadh Agricultural Universities, respectively.

### Selection of experimental animals

A total of 10 healthy Surti and Jafarabadi buffaloes of each breed were selected at random from Livestock Research Station, Navsari and Cattle Breeding Farm, Junagadh, Gujarat, respectively, for this study. All the buffaloes selected under the study belonged to first, second, and third parity. Selected buffaloes were categorized for the ease of data analysis and comparisons into two groups, viz., S60 (Surti buffaloes 60^th^ day pp) and J60 (Jafarabadi buffaloes 60^th^ day pp).

### Sample collection

The milk samples (1500 ml per animal) were collected twice a day on 60^th^ day of lactation relative to parturition. Whole milk sample from each selected animal was collected during milking into a sterile bucket, and milk yield was determined using electronic balance. 50 ml aliquot was taken in polypropylene tube and was subjected to milk composition analysis immediately after collection. The milk was kept at 4°C until processed for MEC purification.

### Milk yield and composition

Test day milk yield (TDMY) in kg was calculated by combining morning and evening milk yield of the collection day (60^th^ day). Cumulative milk yield 60 days pp (CMY60) was calculated by combining TDMY of first 60 days pp, respectively. Milk composition of samples such as percent milk protein, fat, SNF, and lactose was analyzed using Lactoscan milk analyzer (Netco, India) as per manufacturer’s instructions (Table-1). Fat and protein corrected TDMY (FPCTDMY) was calculated by correcting TDMY to a fat percent at 4.0% using the formula:

**Table-1:** Mean milk yield and composition traits of Surti and Jafarabadi buffaloes at day 60^th^ pp.

Traits/groups	S60	J60	t values
N	10	10	
TDMY (kg)	4.92±0.32	5.41±0.56	1.6
FPCTDMY (kg)	5.96^a^±0.40	7.77^b^±0.91	2.82*
CMY60 (kg)	235.55^a^±18.34	310.20^b^±27.43	2.25*
Fat percent	5.73^a^±0.16	7.38^b^±0.41	4.04**
SNF percent	10.11^a^±0.22	10.67^b^±0.12	4.95**
Protein percent	3.52^a^±0.06	3.89^b^±0.05	14.02**
Lactose percent	5.51^a^±0.15	5.83^b^±0.07	3.96**

* Significant at p≤0.05,

** Highly significant at p≤0.01. Means bearing different superscript between groups differed significantly. N=Number of observations, SNF=Solid not fat, TDMY=Test day milk yield, FPCTDMY=Fat and protein corrected test day milk yield, CMY60=Cumulative milk yield 60 days postpartum

FPCTDMY (kg) = TDMY (kg) × [0.337 + (0.116 × fat percent) + (0.06 × protein percent)]

### MEC purification from milk

The milk was filtered through fine muslin cloth to remove particulate impurities and centrifuged at 1800 g for 30 min at 4°C. Fat layer was removed and skimmed milk was decanted carefully without disturbing cell pellet, which was resuspended in phosphate buffered saline (PBS), washed twice in PBS at 1850 g for 15 min and resuspended in 1 ml of 1% PBS-bovine serum albumin (BSA). Dynabeads (Invitrogen, Norway) coated with primary mouse monoclonal anti-cytokeratin 8 antibody were used to purify MEC as described Sigl *et al*. [18] with some modifications. Briefly, the dynabead solution was washed twice with 1 ml of 1% PBS-BSA to remove preservatives. Dynabead suspension was incubated for 10 min anticytokeratin 8 antibody (Thermo Scientific, USA) on a rotary mixer at 4°C. Unbound antibodies were removed, and antibody-coated dynabeads were further resuspended in 1 ml of 1% PBS-BSA. For purification of MEC, 1 ml cell suspension was incubated with 25 µl antibody-bead complex for 20 min on rotary mixer at 4°C. Unbound cells were removed using magnetic stand and finally enriched MEC were resuspended in 1 ml of 1% PBS-BSA. Purified MECs were collected and mixed with ice-cold Trizol (Qiagen, USA) and stored at −80°C until processed for RNA isolation.

### RNA isolation and cDNA synthesis

Total RNA was extracted from purified primary bovine MECs (pBMEC) using Trizol reagent according to the manufacturer’s protocol as adapted from Sigl *et al*. [18]. After extraction, RNA was purified using miRNeasy Mini kit (Qiagen, Germany) and followed by on-column digestion with the RNase-free DNase (Qiagen, Germany) RNA was quantified by Nanodrop spectrophotometric (Thermo Scientific ND 2000C, USA). A260/A280 ratio was 1.8-2.0 for all samples. RNA integrity was confirmed by denaturing agarose gel electrophoresis using the method described by Miller [19]. cDNA was prepared by using first strand cDNA synthesis kit (QuantiTect^®^ Reverse Transcription Kit) according to manufacturer’s instructions.

### Real time quantitative polymerase chain reaction (PCR)

Major genes responsible for the synthesis of fat were investigated for their differential expression along with marker gene for epithelial cells. Relative expression of major milk lipogenic and keratin genes was quantified by real-time PCR (ABi, USA) and analyzed using Applied Biosystems 7500 software v2.0.5. The primers were selected from published references and commercially synthesized from Eurofins Genomics, India. The specificity of primers was checked by NCBI blast program (http://www.ncbi.nlm.nih.gov/BLAST/). The primers used are given in Table-2. All real-time PCR reactions were of 20 µl and consisted of 10 µl of 1× real-time SYBR green PCR master mix (QuantiFast), 2.0 µl cDNA, 1.0 µl each of primers (10 pM), and 6.0 µl water. The PCR protocol involving denaturation step (94°C, 15 s), annealing combined with extension step (60°C, 30 s), cycling program (45 times) followed by melt curve analysis was used for all genes. A single sharp peak in the melt curve analysis and single band in gel electrophoresis indicated specific amplification for each gene primer pair. No template control was also included for each primer assay. Two biological replicates and three technical replicates were used for each sample.

**Table-2:** Primer sequences for PCR amplification, product sizes, accession numbers and references for various genes under study.

Gene	Sequence (5’–3’)	Product size (bp)	Accession No.	References
Major milk fat genes				
*BTN1A1* for	TGTGTTGCTGCTGATAGAGTGTTAG	165	NM_174508	[20]
*BTN1A1* rev	CCTCCAAGTTCCTTTATGGGATTTC			
*SCD* for	CCTGTGGAGTCACCGAACCT	66	AF481915	[21]
*SCD* rev	GTGTTGCCAATGATCAGGAAGA			
*LPL* for	CAGAAGCTCCAAGTCGCCTTT	73	M16966	[21]
*LPL* rev	GACCCCCTGGTGAATGTGTG			
*GPAM* for	GCAGGTTTATCCAGTATGGCATT	68	AF469047Y 284842	[15]
*GPAM* rev	GGACTGATATCTTCCTGATCATCTTG			
*ACACA* for	GAGTTCCTCCTTCCCATCTACCA	123	NM_174224	[22]
*ACACA* rev	GGTGCGTGAAGTCTTCCAATC			
*LPIN* for	GAGGGGAAGAAACACCACAA	195	XM_002707716	[23]
*LPIN* rev	GTAGCTGACGCTGGACAACA			
Marker of epithelial cells							
*KRT8* for	ACTGGCTACGCAGGTGGACT	200		[24]
*KRT8* rev	CCGCAAGAGCCTTTCACTTG			
Reference genes							
*GAPD* for	TGGAAAGGCCATCACCATCT	65	NM_001034034.1	[25]
*GAPD* rev	CCCACTTGATGTTGGCAG			

for=Forward, rev=Reverse, BTN1A1=Butyrophilin subfamily 1 member A1, SCD=Stearoyl-CoA desaturase, LPL=Lipoprotein lipase, GPAM=Glycerol-3-phosphate acyltransferase Mitochondrial, ACACA=Acetyl-coenzyme A carboxylase alpha, LPIN=Lipin, KRT8=Keratin 8, GAPD=Glyceraldehyde-3-phosphate dehydrogenase

### Data normalization and analysis

In this study, the NormFinder algorithm was used. The housekeeping gene glyceraldehyde-3-phosphate dehydrogenase was used as reference index and used for normalization. Quantitative cycle (Cq) values were calculated by Applied Biosystems 7500 software v2.0.5. Relative mRNA levels were then calculated for each gene using ΔΔCq method. Cq of housekeeping genes were subtracted from Cq of each gene to obtain ΔCq [26]. Calibrator ΔCq was subtracted from each sample’s ΔCq, and then relative mRNA value was calculated by the formula 2−ΔΔCq [27].

### Estimation of parameters

#### Statistical analysis

The data on milk yield and milk composition was subjected to statistical analysis using Statistical Package for Social Sciences (Version 20.0) software. Descriptive statistics specifying mean±standard error of mean (SEM), highest and lowest value were calculated for each group. One-way ANOVA procedure was undertaken to compare means. Post-hoc multiple comparisons were made using Duncan multiple new range test. Independent sample t-test was used for two-group comparisons. Bivariate correlations were calculated using Pearson correlation coefficient. The size of correlation (very high, high, moderate, low, and negligible) was interpreted as per the standard classification [28].

## Results and Discussion

### CMY60

The mean±SEM values of CMY60 were 235.55±18.34 and 310.20±27.43 kg in Surti and Jafarabadi buffaloes, respectively (Table-1). The lowest to highest CMY60 observed among Surti and Jafarabadi buffaloes were 145.20-351.60 and 167.70-416.5 kg, respectively. The mean CMY60 of Jafarabadi buffalo was 31.69% significantly (p≤0.05) higher than Surti buffalo.

### Somatic cells, pBMECs and RNA yield

In this study, pBMEC was successfully recovered from milk (1500 ml) using indirect method by antibody-mediated magnetic separation. It has been shown in several studies that milk secreting cells can be purified from milk using immunomagnetic separation [18,29,30]. Exfoliated pBMEC obtained directly from milk in the present study were expected to represent actual gene expression profile at that point of time in mammary glands of respective group. The detailed statistics of somatic cells count (SCC), pBMEC obtained from total somatic cells and RNA yield from pBMEC in milk of Surti and Jafarabadi buffaloes at day 60^th^ pp is presented in Table-3.

**Table-3:** Mean somatic cells, pBMECs and RNA yield among different groups.

Traits/groups	S60	J60	t values
N	10	10	
SCC (×1000/ml)	197.68^b^±4.74	175.38^a^±5.51	9.41**
pBMEC (×1000/ml)	4.99±0.52	5.57±0.45	0.70
pBMEC recovery %	2.54±0.27	3.19±0.28	2.71
RNA yield (µg)	7.56±0.81	6.31±1.09	0.84

*Significant at p≤0.05,

** Highly significant at p≤0.01. Means bearing different superscript between groups differed significantly. N=Number of observations, pBMEC=Primary bovine mammary epithelial cells, SCC=Somatic cells count

### Somatic cell count

Milk SCC depends mainly on immune status of the udder and also on various nongenetic factors. In most developed dairy industries, various regulatory limits have been applied to milk for human consumption. The European Union Directives (92/46CEE and 94/71 CEE) set a limit of 400,000 cells/ml for SCC in raw buffalo milk when the milk is used for products made with raw milk. The SCC observed in this study for all the milk samples was less than the standard set by European Union Directives thus; all the milk samples used in this study were expected to be produced from healthy quarters of buffaloes. In this study, the SCC was found to be higher in Surti buffaloes as compared to Jafarabadi buffaloes. This may be attributed to agro-climatic differences of higher rainfall and humid conditions during the study period in home tract of Surti buffaloes compared to Jafarabadi buffaloes. Same way SCC variation had been noted between breeds of dairy animals. The high-producing cattle breeds such as Brown Swiss (423.31×10^3^ cells/ml) and Black Holstein (310.36×10^3^ cells/ml) have higher presence of SCC/ml in milk compared to other breeds [31]. The overall mean SCC reported in this study for different groups were comparable with the average SCC of 1.99±0.03×10^5^ cells/ml with the range of 1.86×10^5^-2.12×10^5^ cells/ml of milk reported from healthy quarters of buffaloes [32]. Mean SCC of 3.21×10^5^ ±0.18 cells/ml, higher than the present study, had also been reported in milk from normal buffalo [33]. Contrastingly, Kavitha *et al*. [34] observed lower mean normal SCC of 1.6×10^5^ cells/ml in buffalo milk.

### pBMECs recovery

Varying proportion of SCC in the milk had been reported in different species, breeds and at different stages of lactation with or without increased shedding of pBMEC. Hence, varying amount of milk was used to extract purified MEC from milk to analyze mammary transcripts from different species [35]. In this study, the mean pBMEC recovered (×1000/ml milk) from total somatic cells were comparable in S60 (4.99±0.52) and J60 (5.57±0.45) groups. Lower pBMEC that ranged from 1.10±0.06 ×10^3^ to 1.40±0.03 ×10^3^ cells/ml milk at different stage of lactation had also been reported in cows [18]. Thus, it can be concluded that pBMEC count/ml in buffaloes milk under this study is higher as compared to earlier reported pBMEC count/ml in cow milk.

### Primary bovine mammary epithelial recovery percent

The percentages of pBMEC in relation to total milk cells among different groups in the present study were steady with non-significant at 60 pp. Boutinaud *et al*. [29] had isolated lower 1.54×10^3^ pBMEC/ml in Holstein-Friesian cow’s milk around day 162 pp which comprised 2% of total milk cells. Contrary to this, significant differences in percentage of pBMEC in relation to total milk cells were reported in cows [18] whereby, percentage of pBMEC increased between day 8 pp (2.0±0.2%), day 43 pp (5.6±0.8%, p<0.001) and day 57 pp (6.7±1.0%). This may be attributed to species difference in said study. In this study, the mean percent pBMEC recovered from total cells ranged between 2.13% and 3.19% in milk of Surti and Jafarabadi buffaloes. Thus, in this study, pBMEC shed continuously in small proportion with respect to total somatic cells.

### RNA yield

In the present study, the mean RNA yield (µg) of Surti and Jafarabadi buffaloes did not differ at day 60 pp. Similar results were obtained in cows, where extracted quantity of pBMEC mRNA did not vary during experimental timeframe [18]. In the present study, the RNA yield and pBMEC in milk of Surti buffaloes were positively and significantly correlated (0.76). Boutinaud *et al*. [36] reported that total RNA extracted, and the numbers of epithelial cells were significantly and positively correlated (0.67) in goat milk as observed in the present study.

### Relative expression of major lipogenic genes and epithelial cell marker gene

The main focus of the present study was to explore the relative expression of major milk lipogenic genes in Surti and Jafarabadi buffalo breeds at day 60^th^ pp. Usually, the stage of lactation potentially affects relative expression of major milk fat-related genes. In addition, maxima of mRNA abundances were reached during the first 2 weeks of lactation followed by respective significant declines toward day 57 pp in Holstein-Friesian cows [18]. In this study, all the genes were successfully amplified and quantified in total RNA isolated from pBMEC in both Surti and Jafarabadi buffalo breeds. The amplification plots and melt curves were found to be optimum and single band was visualized on agarose gel for all the genes. The mean relative expression “15-ΔCq (log_2_)” values of milk lipogenic genes and keratin gene among different groups have been presented in Table-4. Relative folds increase/decrease between two groups for various gene transcripts against unit folds expression of one rotationally chosen group had been presented in Table-5.

**Table-4:** Mean relative expression of major milk lipogenic genes and keratin gene between groups.

Groups	S60	J60	t value
N	10	10	
*BTN1A1*	3.96±0.10 (−0.57)	3.98±0.13 (−0.73)	0.05
*SCD*	3.60±0.11 (2.83)	3.53±0.15 (3.47)	0.10
*LPL*	3.68±0.12 (2.20)	3.63±0.14 (2.64)	0.01
*GPAM*	3.50±0.12 (3.68)	3.58±0.13 (3.05)	0.32
*ACACA*	3.08±0.17 (6.53)	3.18±0.22 (5.94)	0.35
*LPIN*	3.33±0.12 (4.94)	3.49±0.18 (3.73)	0.78
*KRT8*	3.62±0.06 (2.73)	3.71±0.03 (1.95)	1.63

Values in parenthesis are mean (∆Cq) values. BTN1A1=Butyrophilin subfamily 1 member A1, SCD=Stearoyl-CoA desaturase, LPL=Lipoprotein lipase, GPAM=Glycerol-3-phosphate acyltransferase Mitochondrial, ACACA=Acetyl-coenzyme A carboxylase alpha, LPIN=Lipin, KRT8=Keratin 8

**Table-5:** Folds increase/decrease in major milk lipogenic genes and keratin gene transcripts among different groups.

Groups	A		B	
	
	S60	J60	S60	J60
*BTN1A1*	1	1.12	0.90	1
*SCD*	1	0.65	1.56	1
*LPL*	1	0.74	1.36	1
*GPAM*	1	1.56	0.66	1
*ACACA*	1	1.52	0.66	1
*LPIN*	1	2.33	0.43	1
*KRT8*	1	1.72	0.58	1

A and B represents relative folds increase/decrease in groups for various gene transcripts against unit folds expression of one rotationally chosen group. BTN1A1=Butyrophilin subfamily 1 member A1, SCD=Stearoyl-CoA desaturase, LPL=Lipoprotein lipase, GPAM=Glycerol-3-phosphate acyltransferase Mitochondrial, ACACA=Acetyl-coenzyme A carboxylase alpha, LPIN=Lipin, KRT8=Keratin 8

### Correlations among relative expressions of major milk lipogenic gene transcripts

The correlation coefficients among relative expression “15-ΔCq (log_2_)” values of lipogenic gene transcripts among different groups have been presented in Table-6.

**Table-6:** Correlation coefficients among relative expression of major milk lipogenic genes day 60 pp in Jafarabadi buffaloes (above diagonal) and Surti buffaloes (below diagonal).

Gene transcripts	*BTN1A1*	*SCD*	*LPL*	*GPAM*	*ACACA*	*LPIN*
*BTN1A1*	-	0.64*	0.89**	0.81**	0.87**	0.87**
*SCD*	0.65*	-	0.60	0.60	0.71*	0.66*
*LPL*	0.82**	0.88**	-	0.81**	0.84**	0.79**
*GPAM*	0.91**	0.86**	0.91**	-	0.69*	0.63*
*ACACA*	0.66*	0.84**	0.83**	0.88**	-	0.98**
*LPIN*	0.78**	0.85**	0.84**	0.94**	0.96**	-

* Significant at p≤0.05,

** Highly significant at p≤0.01. BTNA1=Butyrophilin subfamily 1 member A1c, SCD=Stearoyl-CoA desaturase, LPL=Lipoprotein lipase, GPAM=Glycerol-3-phosphate acyltransferase mitochondrial, ACACA=Acetyl-coenzyme A carboxylase alpha, LPIN=Lipin

### Correlations among milk yield, composition and relative expressions of major milk lipogenic genes

The correlation coefficients among milk yield, milk composition and relative expression “15-ΔCq (log_2_)” values of major milk lipogenic genes among different groups have been presented in Tables-7 and 8. The gene-wise comparative results as well as and between breed differences observed in this study are described as follows:

**Table-7:** Correlation coefficients among milk yield, composition and relative expression of major milk lipogenic genes in Surti buffaloes at day 60 pp.

Traits/gene transcripts	*BTN1A1*	*SCD*	*LPL*	*GPAM*	*ACACA*	*LPIN*
CMY60 (kg)	−0.02	0.01	0.08	0.01	0.06	−0.06
TDMY (kg)	0.03	0.04	0.13	0.04	0.03	−0.08
FPCTDMY (kg)	0.10	0.14	0.22	0.17	0.19	0.07
Fat percent	0.22	0.38	0.36	0.46	0.57	0.49
SNF percent	0.04	−0.32	−0.35	−0.14	−0.18	−0.06
Protein percent	0.12	−0.25	−0.28	−0.02	−0.04	0.08
Lactose percent	0.01	−0.35	−0.37	−0.20	−0.25	−0.13

*Significant at p≤0.05,**Highly significant at p≤0.01. TDMY=Test day milk yield, FPCTDMY=Fat and protein corrected test day milk yield, CMY60=Cumulative milk yield 60 days postpartum, SNF=Solid not fat, BTNA1=Butyrophilin subfamily 1 member A1c, SCD=Stearoyl-CoA desaturase, LPL=Lipoprotein lipase, GPAM=Glycerol-3-phosphate acyltransferase mitochondrial, ACACA=Acetyl-coenzyme A carboxylase alpha, LPIN=Lipin

**Table-8:** Correlation coefficients among milk yield, composition and relative expression of major milk lipogenic genes in Jafarabadi buffaloes at day 60 pp.

Traits/gene transcripts	*BTN1A1*	*SCD*	*LPL*	*GPAM*	*ACACA*	*LPIN*
CMY60 (kg)	0.43	0.48	0.40	0.65*	0.22	0.19
TDMY (kg)	0.17	0.28	0.16	0.36	0.13	0.12
FPCTDMY (kg)	0.20	0.32	0.26	0.47	0.21	0.19
Fat percent	0.20	0.18	0.43	0.49	0.38	0.34
SNF percent	−0.30	−0.37	−0.41	0.00	−0.57	−0.56
Protein percent	−0.23	−0.54	−0.37	−0.03	−0.56	−0.54
Lactose percent	−0.36	−0.65*	−0.49	−0.19	−0.67*	−0.65*

* Significant at p≤0.05,

**Highly significant at p≤0.01. TDMY=Test day milk yield, FPCTDMY=Fat and protein corrected test day milk yield, CMY60=Cumulative milk yield 60 days postpartum, SNF=Solid not fat, BTNA1=Butyrophilin subfamily 1 member A1c, SCD=Stearoyl-CoA desaturase, LPL=Lipoprotein lipase, GPAM=Glycerol-3-phosphate acyltransferase mitochondrial, ACACA=Acetyl-coenzyme A carboxylase alpha, LPIN=Lipin

#### BTN1A1 relative expression

The mean relative expression of *BTN1A1* did not differ between Surti and Jafarabadi buffaloes at day 60^th^ pp (1.12-fold). Thus, it can be said that relative expression of *BTN1A1* (regulating lipid droplet formation) was not affected significantly by breed of buffaloes taken in the present study. Wickramasinghe *et al*. [9] studied gene expression in somatic cells in Holstein cows using RNA-Seq technology to examine the genes expressed in transition (day 15), peak (day 90) and late (day 250) lactation. In contrast to the present study, *BTN1A1* showed higher expression in transition lactation milk somatic cells (MSCs) and a significant decrease in the expression levels was observed during the course of lactation. In another study, *BTN1A1* followed the similar trend with high expression in early followed by mid and late lactation stages in milk epithelial cells of Murrah buffaloes. Genes encoding *BTN1A1* proteins showed higher expression in transition lactation MSC, and a significant decrease in the expression levels for *BTN1A1* were observed during the course of lactation. In Sahiwal cows, the expression was almost equally comparable with all the stages of lactation [37]. Wu *et al*. [38] studied the expression pattern of *BTN1A1* in 10 tissues of water buffalo. They reported abundant expression of *BTN1A1* in mammary gland, trace expression in intestine, pituitary, brain, abomasum, kidney, liver and muscle, and no expression in heart and lung. In the present study, significantly high correlations were observed for relative expression of *BTN1A1* with relative expressions of *SCD*, *LPL*, *GPAM, ACACA* and *LPIN* in the S60 and J60 groups. There were no significant correlations between transcript abundance of *BTN1A1* and milk composition traits among various groups to draw out any conclusion.

#### SCD relative expression

Like *BTN1A1*, there was no significant change in expression of *SCD* between Surti and Jafarabadi buffaloes at day 60 pp (0.65-fold). Bionaz and Loor [39] observed >40-fold up-regulation of *SCD* at peak lactation (60 day pp) with subsequent decline in late lactation in Holstein-Friesian cattle, which agrees with the suggestion by Kinsella, based on lactating mammary activity [40], that *SCD* plays a key role in TG synthesis. Yadav *et al*. [25] found non-significant difference in the expression of *SCD* in lactating and nonlactating buffalo. Han *et al*. [41] reported the >8-fold upregulation in *SCD* expression during lactation in mouse mammary gland. In another study, *SCD* followed the similar trend with high expression in early (0-20 days) followed by mid (90-130 days) and late (>240 days) lactation stages in milk epithelial cells of Murrah buffaloes. In Sahiwal cows, in accordance to our study, the expression was almost equally comparable with all the stages of lactation [37]. Yadav *et al*. [42] reported contrasting results in Murrah buffaloes compared to our study. The mRNA expression of *SCD* was upregulated (~5-fold) during peak lactation (60 days pp) where highest mRNA level of *SCD* was observed, which further decreased to basal level at 90 days pp lactation until late lactation. In the present study, while analyzing the data for correlation among relative abundance of major milk fat gene transcripts, significant to highly significant correlation were observed for relative expression of *SCD* with *LPL, ACACA*, and *LPIN* in S60 group. Positive correlation of relative expression of *SCD* existed with *ACACA* and *LPIN* in S60 and J60 groups. The relative expression of *SCD* was highly significant and negatively correlated with lactose percent in J60 group. In accordance to our study, strong correlation was reported between *SCD* and *ACACA* (0.81) in Holstein–Friesian cows [43]. Yadav *et al*. [42] however, reported contrasting results in Murrah buffaloes in which *SCD* showed weak correlation with *LPL* (0.07), *ACACA* (−0.12) and fat yield (−0.47). Strong correlation was observed between *SCD* with milk yield (0.53) and *LPIN* (0.80).

#### LPL relative expression

The relative transcript abundance of *LPL* was found to be steady in between two groups under study. There were no significant differences in expression of *LPL* between Surti and Jafarabadi buffaloes at 60^th^ pp (0.74-fold). Thus, it can be said that relative expression of gene *LPL* was not affected by breed of buffaloes taken in the present study. Bionaz and Loor [39] observed up-regulation in *LPL* at peak lactation (60 days pp) with subsequent decline in late lactation in Holstein-Friesian cattle. The *LPL* expression pattern was similar to the lactation curve. Wickramasinghe *et al*. [9] reported *LPL* showed higher expression in transition (day 15) and peak lactation (day 90) in MSCs of Holstein cows, contrasting present findings. Yadav *et al*. [42] reported contrasting results in Murrah buffaloes in which *LPL* mRNA level showed up-regulation during early lactation (45 day pp) up until peak lactation and then remained unchanged throughout lactation. In the present study, high significant positive correlations between relative expression of *LPL* and other lipogenic genes, i.e., *GPAM*, *ACACA*, and *LPIN* were observed among all the groups. As per Yadav *et al*. [42] *LPL* showed weak correlation with *SCD* (0.07)*, LPIN* (0.38), milk yield (0.17) and fat yield (−0.08), contrasting present findings and moderate correlation with *ACACA* (−0.58).

#### GPAM relative expression

The relative expression of *GPAM* was not affected significantly by breed of buffaloes taken in the present study. Bionaz and Loor [39] reported the expression of *GPAM* mRNA increased by ~10-fold by 60^th^ day pp in Holstein-Friesian cattle. *GPAM* expression agrees with the greater enzyme activity in mammary gland during lactation in non-ruminants [44] and confirms its crucial role in TG synthesis [45]. Wickramasinghe *et al*. [9] reported higher expression of *GPAM* in transition (day 15) followed by a progressive decrease in expression along the lactation in MSCs of Holstein cows. The expression level of *GPAM* were significantly higher in early (0-20 days) compared to mid (90-130 days) and late (>240 days) lactation stages in MSCs of Murrah buffaloes and Sahiwal cows [46]. In the present study, significant positive correlation was observed between relative expression of *GPAM* with *ACACA* and *LPIN* in S60 and J60 groups. *GPAM* was significantly and positively associated with CMY60 in J60 group.

#### ACACA relative expression

The relative transcript abundance of *ACACA* was found to be steady in between two groups under the study. Bionaz and Loor [39] observed up-regulation of *ACACA* at peak lactation (60^th^ day pp) compared to early lactation stages with subsequent decline in late lactation in Holstein-Friesian cattle. [9] reported that, *ACACA* showed a significant decrease in expression along the course of lactation expression in MSCs in Holstein cows. Yadav *et al*. [42] reported *ACACA* showed high expression during early lactation in Murrah buffaloes. However there was slight decrease in mRNA level during 30-45 day pp which further increased at peak lactation, followed by sharp decline at 90 days. In the present study, significant positive correlation was observed between relative expression of *ACACA* and *LPIN* in all the groups. In J60 group, correlation of relative expression of *ACACA* with lactose percent (−0.67) was significant and negative. Yadav *et al*. [42] reported that expression pattern of *ACACA* was negatively correlated with milk yield (−0.88) and positively correlated with fat yield (0.62). *ACACA* showed weak correlation with *SCD* (−0.12) and moderate correlation with *LPL* (−0.58) and *LPIN* (−0.58).

#### LPIN relative expression

The relative abundance of *LPIN* was found to be steady across all the groups under study signifying no significant effect of breed. Bionaz and Loor observed ~20-fold up-regulation of *LPIN* mRNA during lactation in bovine [39]. But contrasting reports were obtained during late lactation, while *LPIN* mRNA was upregulated significantly in mouse; it was downregulated in bovine during late lactation [47]. Wickramasinghe *et al*. [9] reported *LPIN* showed higher expression in transition (day 15) and then a progressive decrease in expression along the lactation in MSCs of Holstein cows. Yadav *et al*. [42] studied the expression pattern of *LPIN* in Murrah buffalo. *LPIN* expression was upregulated at peak lactation (~4-fold) compared with early lactation in buffalo MEC; thereafter, there was a gradual and significant decline in *LPIN* mRNA levels after peak lactation (60 day pp). Decline in *LPIN* mRNA during late lactation, is a trend similar to that reported in bovine. In the present study, a significant positive correlation was observed between relative expression of *LPIN* with *LPL* and *ACACA* in all the groups. All the correlations of relative expression of *LPIN* gene with milk yield and composition traits were not significant except the one with lactose percent in J60 group. Yadav *et al*. [42] reported similar findings in Murrah buffalo that *LPIN* has strong positive correlation with *SCD* (0.80) and *LPL* (0.38) but negative correlation with *ACACA* (−0.57). *LPIN* expression pattern has strong positive correlation with milk yield (0.75) and negative correlation with fat yield (−0.72) across lactation in buffalo.

#### Keratin 8 (KRT8) relative expression (epithelial cell marker)

The relative expression of *KRT8* was almost similar in all the groups. It was almost constantly expressed irrespective of breed and stage of lactation. This might be the result of common source of RNA obtained under the present study exclusively from pBMEC. The epithelial keratins had earlier been also found to be useful markers for epithelial cells [48]. Transcript abundance of *KRT8* was also found to be constant earlier by Sigl *et al*. [18] at different lactation stages in Holstein-Friesian cows.

## Conclusion

The pBMEC can be successfully recovered from 1500 ml of milk of Surti and Jafarabadi buffaloes using antibody-mediated magnetic bead separation as adapted from Sigl *et al*. [18] with slight modifications. The recovered pBMECs further used for recovering RNA for down step quantification of major milk lipogenic gene expression. In this study overall, the relative expression of the six major milk lipogenic genes *BTN1A1*, *SCD*, *LPL*, *GPAM*, *ACACA*, and *LPIN* did not show changes in expression patterns in both Surti and Jafarabadi buffaloes, suggesting expression levels of lipogenic genes are maintained almost uniform till peak lactation without any significant difference. The mean relative expression of *KRT8* gene was almost comparable among all the groups under this study. The genes involved in various interrelated processes of milk fat synthesis such as mammary FA uptake from blood (*LPL*), *de novo* FA synthesis (*ACACA*), desaturation (*SCD*), triacylglycerol synthesis (*GPAM* and *LPIN*), and lipid droplet formation *(BTN1A1*) had also shown positively correlated expression in present study. Out of all the genes *SCD*, *ACACA* and *LPIN* which are involved in *de novo* milk fat synthesis are strongly correlated. However, the mechanism controlling milk fat secretion is quite complex and it is not known whether or not the secretion is constitutive or is regulated. The complexity of mammary molecular adaptations over time can be underscored by gene network analysis as well as the apparent interrelationships that must coordinate the overall process of milk fat synthesis and secretion.

## Authors’ Contributions

MJ, VK, BB and UR designed the study. The experiment was done by MJ, GP, KT and UR whereas laboratory work was done by MJ, GP and KT. All the authors participated in data analysis, draft, and revision of the manuscript. All authors read and approved the final manuscript.
